# Whole-Genome Sequencing of the Tropical Marine Bacterium Nocardiopsis dassonvillei NCIM 5124, Containing the Ectoine Biosynthesis Gene Cluster *ectABC*

**DOI:** 10.1128/mra.00435-22

**Published:** 2022-09-26

**Authors:** Pratik Kadam, Swapnil Kajale, Avinash Sharma, Dhiraj Dhotre, Vitthal Barvkar, Yogesh Shouche, Smita Zinjarde

**Affiliations:** a Department of Biotechnology (with jointly merged Institute of Bioinformatics and Biotechnology), Savitribai Phule Pune University, Pune, India; b National Center for Microbial Resource (NCMR), National Center for Cell Science (NCCS), Pune, India; c Department of Botany, Savitribai Phule Pune University, Pune, India; Indiana University, Bloomington

## Abstract

The genome sequence (7,057,619 bp; GC content, 72.07%) of a tropical marine isolate, Nocardiopsis dassonvillei NCIM 5124, containing the biomedically and biotechnologically important gene cluster *ectABC* is reported here.

## ANNOUNCEMENT

The genus *Nocardiopsis* of the order *Actinomycetales* was first described by Meyer in 1976 (1). The members of this genus were later included in a new family, *Nocardiopsaceae* (2). *Nocardiopsis* species are aerophilic, Gram-positive, non-acid-fast, catalase-positive actinomycetes, with colony characteristics similar to those of *Nocardia* and *Actinomadura* species (3). *Nocardiopsis* spp. have been isolated from saline habitats and produce a variety of bioactive compounds (4–7). A strain of Nocardiopsis dassonvillei isolated from oil-contaminated seawater (deposited in the National Collection of Industrial Microorganisms, India, as NCIM 5124), capable of degrading hydrocarbons, producing proteases, and mediating the synthesis of gold nanoparticles, was used in this study (8–11).

The culture was grown on glucose-yeast-malt extract (GYM) agar medium and incubated at 30°C for 48 h. DNA was extracted using the method described by Yeates et al. (12). The modified method is as follows: a single colony was suspended in 500 μL of extraction buffer (100 mM Tris-HCl [pH 8.0], 100 mM Na_2_EDTA [pH 8.0]), followed by bead beating for 2 to 3 min with glass beads. Proteinase K (NitroGen, USA) was added (20 mg/mL), and the culture was incubated at 55°C for 2 h with intermittent shaking. An aliquot (100 μL) of NaCl (0.5 M) was added, and the culture was incubated at 72°C for 30 min. DNA was extracted using phenol:chloroform:isoamyl alcohol (25:24:1), washed twice with 70% ethanol, dissolved in 1,000 μL Tris-EDTA buffer (pH 8.0), analyzed by electrophoresis (0.8% agarose gel), and visualized by ethidium bromide staining using a UV transilluminator.

Library preparation for Illumina was conducted using the Nextera DNA Flex library preparation kit (Illumina Inc., San Diego, CA, USA). Genomic DNA was sequenced on the Illumina MiSeq platform using paired-end (2 × 250-bp) technology with v2 Illumina chemistry (13, 14). The genome quality was evaluated using the FastQC v0.11.9 tool (15); the raw reads were assembled using the Unicycler v0.4.8 assembler and polished using Pilon v1.23 in the PATRIC v3.6.12 online server (16). Genome finishing was performed using the MeDuSa Web server (17). The quality of the assembly was checked using the tools QUAST v5.1.0rc1 (18) and CheckM v1.2.0 (19). The genome was annotated using the NCBI Prokaryotic Genome Annotation Pipeline (PGAP) v6.1 (20). In all, 1,001,347 reads and 483,998,405 bases were generated, with 70% genome coverage. Default parameters were used for all software unless otherwise noted.

The total length of the genome sequence was 7,057,619 bp; sequencing yielded 35 contigs (*N*_50_, 6,954,860 bp), with a GC content of 72.07% and 97.25% completeness. Among the 6,390 total genes, 6,328 coding sequences and 6,130 proteins were identified. In addition, 57 tRNAs and 2 rRNAs (one 16S rRNA and one 23S rRNA) were found. The genome harbors genes coding for CRISPR arrays, virulence factors, transporters, drug targets, antibiotic resistance, and ectoine biosynthesis. The genome shows the presence of the *ectABC* gene cluster, which is involved in the synthesis of ectoine, a commercially valuable compatible solute (21–25).

### Data availability.

This whole-genome shotgun sequencing project has been deposited at DDBJ/ENA/GenBank under the accession number JALPTI000000000. The version described in this paper is version JALPTI000000000.1. The associated BioProject and Sequence Read Archive accession numbers are PRJNA818875 and SRR19025777.
